# P-1858. Exploring the Role of Fatty acid synthase (FASN) Inhibition in Interferon Gamma Mediated Killing of *Histoplasma capsulatum*

**DOI:** 10.1093/ofid/ofae631.2019

**Published:** 2025-01-29

**Authors:** Resmi Nair, William Buesing, Diego Rossi, George S Deepe

**Affiliations:** MIDC, Tempe, Arizona; University of Cincinnati, Cincinnati, Ohio; University of Cincinnati, Cincinnati, Ohio; University of Cincinnati, Cincinnati, Ohio

## Abstract

**Background:**

*Histoplasma capsulatum* (*Hc*) is the leading cause of fungal respiratory infections in the US. Most cases are mild but 20% require hospitalization. Immunosuppression is a major risk factor for disseminated histoplasmosis. The cytokine Interferon gamma (IFN)-γ is crucial for host resistance to *Hc.* The absence of IFN-γ or its failure to signal affects the host immune response.

Activated CD4^+^ T cells release IFN-γ to arm macrophages(MΦ) to kill intracellular yeasts. Immunometabolism is a key element in the function of MΦ and fatty acid synthase (FASN) regulates inflammatory responses by these cells. FASN is a large multi-enzyme complex comprised of 6 separate enzymatic grooves that work together to produce 16-carbon chain saturated fatty acid (FA), palmitate, from acetyl-coenzyme A (CoA) and malonyl-CoA(Figure 1). The influence of FASN in the IFN-γ-mediated activation of MΦ remains unclear.

**Figure:**
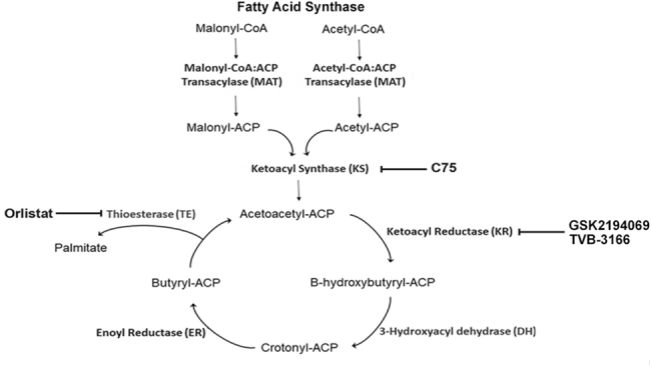

**Key inhibitor targets in FASN complex**

**Methods:**

Figure 1 shows key inhibitor targets in FASN complex. Bone marrow derived Mφ were used in all experiments with a 2 to 1 yeast to Mφ ratio. IFN-γ induced killing of *Hc* in mouse Mφ require 48h. We used the tetrazolium salt XTT to assess the *Hc* viability as this correlates with the colony counts. Controls (*Hc* in Mφ) are set to 100 %. Toxicity of each inhibitor to *Hc* and Mφ was studied first.

Mφ were treated with IFN-γ 4hrs before infection and then treated with graded amounts of FASN inhibitors C 75 (inhibits ketoacyl synthase), orlistat (inhibits thioesterase), and TVB-3166 or GSK2194069 (both inhibit ketoacyl synthase) 2hrs before infection with 2x *Hc* for 48hrs. Intracellular yeast survival at 48hrs was assessed.

**Figure:**
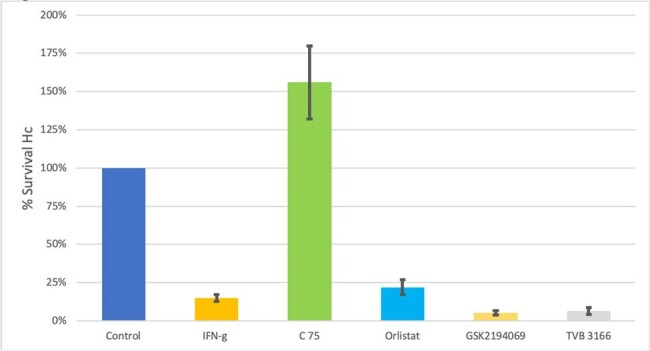

Effect of FASN inhibitors on IFN- γ mediated killing of Hc at 48 hrs.

n= 3-10 , mean ±SEM

**Results:**

Pretreatment with the FASN inhibitor C75 reversed the killing activity of IFN- γ. Neither orlistat or inhibitors of ketoacyl reductase modified the impact of IFN-γ. (Figure 2)

**Conclusion:**

The FASN inhibitor C75 reversed the activity of IFN-γ in killing *Hc*. These results suggest that cholesterol synthesis may be involved in the action of IFN- γ.

**Disclosures:**

**All Authors: No reported disclosures**

